# Cilostazol-Loaded Poly(ε-Caprolactone) Electrospun Drug Delivery System for Cardiovascular Applications

**DOI:** 10.1007/s11095-017-2314-0

**Published:** 2018-01-16

**Authors:** Marek Rychter, Anna Baranowska-Korczyc, Bartłomiej Milanowski, Marcin Jarek, Barbara M. Maciejewska, Emerson L. Coy, Janina Lulek

**Affiliations:** 1 Department of Pharmaceutical Technology, Faculty of Pharmacy, Poznan University of Medical Sciences, Grunwaldzka 6, 60-780 Poznan, Poland; 2 NanoBioMedical Center, Adam Mickiewicz University Poznan, Umultowska 85, 61-614 Poznan, Poland; 3 Department of Macromolecular Physics, Faculty of Physics, Adam Mickiewicz University, Umultowska 85, 61-614 Poznan, Poland

**Keywords:** cilostazol, drug delivery system, electrospinning, polycaprolactone, tissue regeneration

## Abstract

**Purpose:**

The study discusses the value of electrospun cilostazol-loaded (CIL) polymer structures for potential vascular implant applications.

**Methods:**

Biodegradable polycaprolactone (PCL) fibers were produced by electrospinning on a rotating drum collector. Three different concentrations of CIL: 6.25%, 12.50% and 18.75% based on the amount of polymer, were incorporated into the fibers. The fibers were characterized by their size, shape and orientation. Materials characterization was carried out by Fourier Transformed Infrared spectroscopy (FTIR), Raman spectroscopy, differential scanning calorimetry (DSC) and X-ray diffraction (XRD). *In vitro* drug release study was conducted using flow-through cell apparatus (USP 4).

**Results:**

Three-dimensional structures characterized by fibers diameter ranging from 0.81 to 2.48 μm were in the range required for cardiovascular application. DSC and XRD confirmed the presence of CIL in the electrospun fibers. FTIR and Raman spectra confirmed CIL polymorphic form. Elastic modulus values for PCL and the CIL-loaded PCL fibers were in the range from 0.6 to 1.1 GPa. The *in vitro* release studies were conducted and revealed drug dissolution in combination with diffusion and polymer relaxation as mechanisms for CIL release from the polymer matrix.

**Conclusions:**

The release profile of CIL and nanomechanical properties of all formulations of PCL fibers demonstrate that the cilostazol loaded PCL fibers are an efficient delivery system for vascular implant application.

**Electronic supplementary material:**

The online version of this article (10.1007/s11095-017-2314-0) contains supplementary material, which is available to authorized users.

## Introduction

Despite a number of preventive programs and initiatives, atherosclerosis of blood vessels, an underlying cause of a number of cardiovascular diseases, still represents a significant problem among patients of both developed and developing countries. Advanced cases of coronary artery disease or peripheral vascular disease often require surgical intervention, which in severe cases demands vascular graft implantation. Currently, commercially-available artificial grafts are most frequently made of synthetic polymers such as expanded polytetrafluroethylene (ePTFE) and poly-ethylene terephthalate (PET) (1). Despite their broad use in clinical practice, the application of synthetic compounds can be troublesome, as their conduits are susceptible to more frequent thrombosis, neointimal hyperplasia and infections (2,3). This, in turn, may lead to unavoidable surgical re-intervention, even resulting in graft explantation.

Recent years have seen the emergence of technologies of cellular and acellular tissue-engineered vascular grafts (TEVG) formation (4). These biodegradable grafts are implantable prostheses acting as scaffolds; they are degraded gradually over time and replaced with vascular tissue, thus allowing the regeneration of a native blood vessel.

In order to design a more efficient vascular graft, several requirements should be met. The applied materials should provide sufficient mechanical support and degrade gradually, thus permitting vascular conduit regeneration (5). Moreover, the graft should also be able to cope with thrombosis, re-endothelialization and neointimal hyperplasia. A viable TEVG candidate should also be readily available for use, with a cost-effective production method that can be scaled-up to an industrial level (6).

Electrospinning is a versatile technique enabling a facile production of nano- and microfibers from various materials including bioresorbable and biodegradable polymers with great potential in biomimicry (7). This term, in the case of electrospun TEVG, relates not exclusively to vascular tube creation but also to the fact that the size and mechanical properties of nano- and microfibers resemble those of the extracellular matrix (ECM), allowing them to replicate the function of endogenous ECM after implantation (8).

Polycaprolactone (PCL) is a hydrophobic, semicrystalline polymer characterized by slow degradation time and low toxicity (9). PCL is often used in electrospun scaffold materials for cardiovascular application due to its favorable mechanical properties (10). PCL-based grafts have demonstrated structural integrity and graft patency for a period of seven weeks *in vivo* (11), and have been found to be suitable substitutes in an abdominal aortic replacement in an *in vivo* rat model, presenting rapid endothelialization and good mechanical properties for up to 18 months (12).

In addition, the high surface area and loading capability of electrospun grafts made of nano- and microfibers expand their potential application, as they can be utilized as a novel form of drug delivery system (DDS). By incorporating various active pharmaceutical ingredients (API), this approach addresses issues connected with acute thrombosis or problems of maintaining cellular homeostasis in the region of graft implantation: insufficient endothelialization followed often by neointimal hyperplasia which can result in graft failure (13,14).

To address these issues, an effective antiplatelet agent, acetylsalicylic acid (ASA) has been used to improve the hemocompatibility of electrospun vascular grafts and limit thrombus formation (15). Paclitaxel (PTX), an antiproliferative agent widely used in drug-eluting stents to prevent neointimal hyperplasia, has also been incorporated in electrospun PCL grafts to limit vascular smooth muscle cells (VSMC) proliferation (16). Antithrombotic agents including dipyridamole (DPA) have also been incorporated in electrospun vascular grafts to address the issue of neointimal hyperplasia by reducing the VSMC proliferation and promoting the re-endothelialization of graft lumen, thus reestablishing vascular homeostasis (17).

Cilostazol (CIL) and its several metabolites act as a phosphodiesterase III inhibitors, which after *per os* administration suppress platelet aggregation (18). CIL is classified as class II in biopharmaceutical classification system (BCS), due to its high permeability and low aqueous solubility. Recently, CIL was found to inhibit vascular graft stenosis by reducing VSMC proliferation and local inflammation *in vivo* more effectively than acetylsalicylic acid (19). In another clinical study, CIL was shown to influence vascular and angiogenesis biomarkers recruiting endothelial progenitor cells, thus restoring injured endothelium (20).

CIL physicochemical properties and biochemical influence on the vascular homeostasis makes it a potential candidate for incorporation into new materials for cardiovascular application. The current trend in newly developed materials for cardiovascular implants design involves a broad use of biodegradable polymers (21). When designing a product with drug-eluting capabilities it is, therefore, crucial to extensively investigate drug-polymer interactions and fully characterize physicochemical properties of produced materials. However, in the case of CIL, a scarce number of such approaches have been reported and none regarding the electrospinning technique. One of these reports has investigated CIL interactions with polymers used in oral dosage forms development as its main aim was to improve CIL solubility and bioavailability after *per os* administration (22). Only one report has shown that CIL incorporation is possible when directly blended with a biodegradable polymer used in cardiovascular implant development (23). Moreover, study conducted by Parker *et al*. demonstrated that antiplatelet agent like CIL can mitigate vascular implant thrombosis. However, physicochemical properties of CIL and biodegradable polymer blend was reported to a marginal extent. Furthermore, use of a low-volatile solvent hampered the solidification process of drug-loaded polymer reservoirs. During the production process, CIL as-received polymorphic form A was changed to form B, which may spontaneously convert overtime back to form A thus affecting the release kinetics from polymer system (24).

The aim of the present work was to incorporate the recently-discovered properties of CIL and the advantages of electrospun materials in the fabrication of a drug-delivering vascular graft for the cardiovascular application. Our study demonstrates the incorporation of the hydrophobic drug - cilostazol (CIL) into the PCL fibers produced by electrospinning technique. It describes the production of fast-solidifying PCL fibers containing CIL in various amounts. CIL-loaded electrospun PCL structures were prepared and extensively characterized. Our study demonstrates no change in CIL polymorphic form after electrospinning process. The study evaluates the potential of electrospun PCL-based materials as novel DDS for the cardiovascular application.

## Experimental

### Production of PCL and PCL + CIL Fibrous Structures

**Fig. 1:**
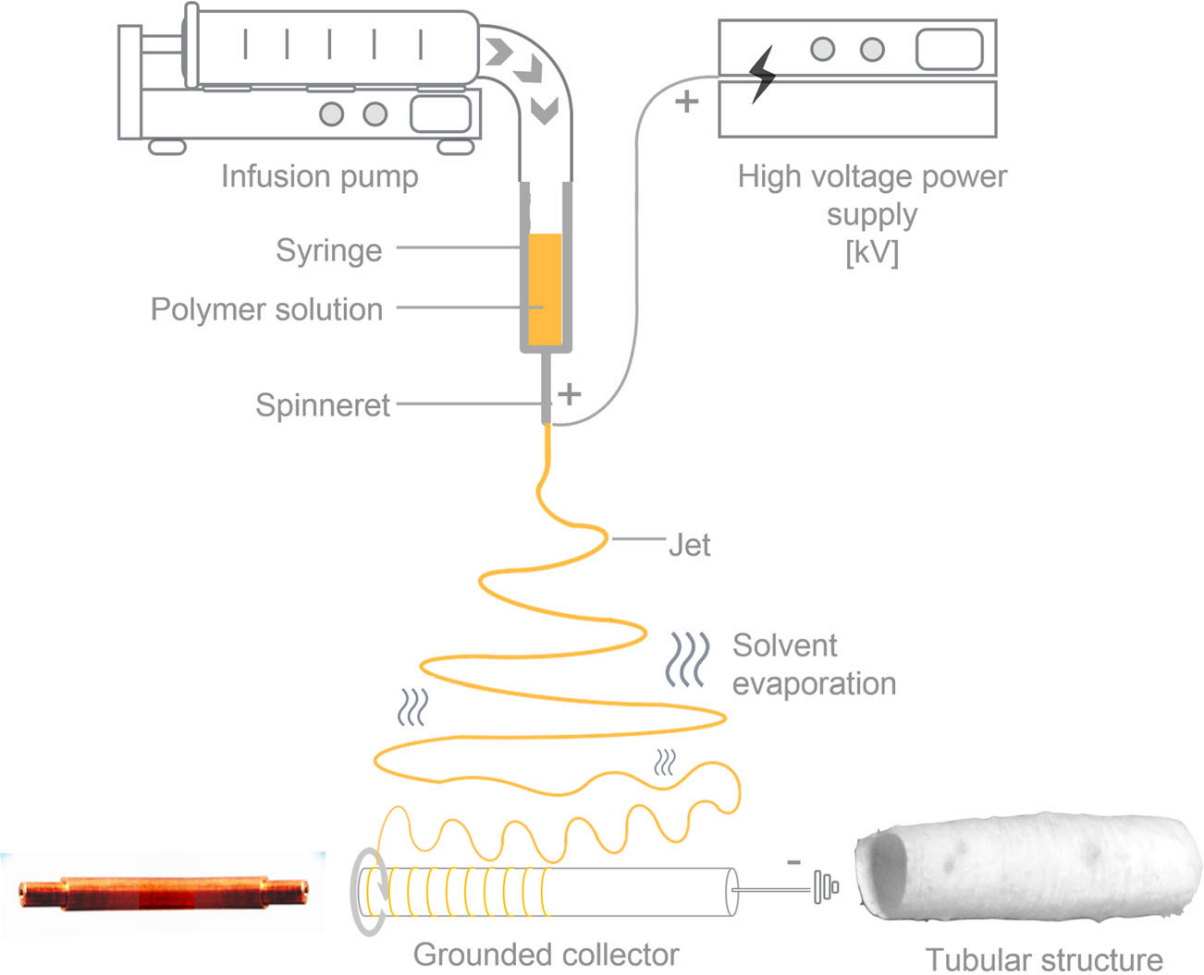
Scheme of an electrospinning setup for the fabrication of PCL fibers.

Poly(ε-caprolactone) (PCL) and composite PCL + cilostazol (CIL) fibers were produced using the electrospinning technique (Fig. 1). The starting solution for the synthesis was prepared in a few steps. Firstly, PCL mixture was obtained at different concentrations of 6%, 8% and 10% (wt) in a 3:1 (*v*/v) mixture of chloroform and methanol. In order to incorporate active substance, direct blending technique was used. CIL was added to the PCL mixtures in three different concentrations of 6.25%, 12.50% and 18.75%, the percentage of the drug was calculated as a dry amount of CIL to PCL in a final product. To obtain concentration of 6.25% CIL, 37.5 mg, 50.0 mg, 62.5 mg of CIL were added to 10 g of 6%, 8% and 10% PCL solutions, respectively. In case of 8% PCL formulation, 100 mg and 150 mg of CIL were added, to obtain concentration of 12.50% and 18.75% CIL based on the amount of polymer.

The PCL solutions, and then the PCL + CIL solutions, were stirred and left to obtain homogeneous mixtures. Following this, 1 ml amounts of the solutions were taken to produce the electrospun samples. A rotating drum collector, 40 mm long and 8 mm in diameter, was then used to obtain fibrous, tubular structures suitable for potential vascular graft applications. The drum collector was covered with aluminum foil to allow the removal of the three-dimensional tubular structures (Fig. 1).

A non-rotating metallic plate collector was also used to obtain and study single fibers. The collecting speeds were set at 0 rpm for the stationary collector and 1000, 2000 and 4000 rpm for the rotating drum collector. The distance between the needle and the collector was 15 cm and the applied voltage was 10 kV. The solutions were added to the process at a constant flow rate of 2 ml/h.

PCL of 80 kDa molecular weight was purchased from Sigma-Aldrich. Cilostazol was obtained from Lekam Pharmaceutical Company Ltd. (Poland).

### Electrospinning Efficiency

After electrospinning, the fibrous structures were removed from the collector as tubular structures. The electrospun tubes were weighted to calculate the efficiency of the electrospinning process, based on the following equation:

1 $$ \mathrm{Electrospinning}\kern0.5em \mathrm{efficiency}\left(\%\right)=\frac{{\mathrm{m}}_{\mathrm{obt}}}{{\mathrm{m}}_{\mathrm{exp}}}\times 100 $$

where m_obt_ represents the mass of the obtained structure, and m_exp_ is the total sum of the masses of the polymer and drug used for the preparation of the structures, excluding solvents, which evaporate during the production process. Generally, electrospinning utilizes organic solvents which due to their volatility, evaporate very quickly during the process. Additionally, during the elongation phase of electrospinning process, the location of charge in the polymer jet starts to change, which results in a number of bending instabilities, causing a reduction of jet diameter on its way from the spinneret to the collector (Fig. 1). This elongated path increases evaporation time of volatile organic solvents thus contributing to the collection of solidified product on the grounded collector (25).

### Entrapment Efficiency

Entrapment efficiency was determined by UV-Vis spectrophotometry at a characteristic wavelength of CIL (λ_max_ = 257 nm). PCL did not interfere with the analysis as it showed no signal at this wavelength. A known mass of electrospun tubular structure was dissolved in chloroform to destroy the structure and release the CIL. The amount of CIL was calculated from the calibration curve in the range of analyte concentrations between 0.1 mg/mL and 0.8 mg/mL (A = 4.0449*c - 0.116; R^2^ > 0.99). The entrapment efficiency was calculated as:

2 $$ \mathrm{Entrapment}\kern0.5em \mathrm{efficiency}\left(\%\right)=\frac{{\mathrm{drug}}_{\mathrm{obt}}}{{\mathrm{drug}}_{\mathrm{exp}}}\times 100 $$

drug_obt_ represents the weight of the drug determined in obtained structure, and drug_exp_ is the weight of the drug expected in obtained structure, based on the known electrospinning efficiency.

### Scanning Electron Microscopy (SEM) Studies

The morphological properties of the fibers were analyzed using a scanning electron microscope (SEM, Jeol 7001TTLS) with an accelerating voltage of 1.0–5.0 kV. The electrospun samples were coated with a thin gold layer to render them electrically conductive prior to microscopy. Both the inner and outer sides of the polymer mats produced by electrospinning on the rotating collector were examined. Micrographs of nearly 100 fibers were randomly selected, and their average diameter was measured using Fiji software (26). In addition, the fiber orientation coherence was measured using the Orientation J plugin, which automates the orientation analysis (27,28). Quantitative orientation measurements were conducted for 50 regions of SEM micrographs of electrospun samples based on the evaluation of the structure tensor in a local area. The coherency coefficient values were described in the range of 0 to 1, where an increasing value corresponded to greater fiber alignment. Visual orientation analysis was conducted by OrientationJ and involved the structure tensor calculations for each pixel in the SEM image. The local orientation properties were computed and then visualized as a color image with the fiber orientation being encoded in the color.

### Hygroscopicity Studies

Hygroscopicity was determined according to the European Pharmacopeia (29). The electrospun samples were placed in a climatic cabinet set at 25°C with a relative humidity of 80 ± 2%. The humidity and temperature in the cabinet were kept constant. Empty glass vessels (m_1_) and vessels containing electrospun PCL tubes with and without CIL load were weighed (m_2_). Samples were prepared in triplicate. Unstoppered vessels were placed in the cabinet for 24 h. After 24 h of storage, the samples were removed from the cabinet, and the mass of each sample was measured (m_3_). The percentage increase in mass was calculated by the following expression:

3 $$ \mathrm{mass}\  \mathrm{increase}\left(\%\right)=\frac{{\mathrm{m}}_3-{\mathrm{m}}_2}{{\mathrm{m}}_2-{\mathrm{m}}_1}\times 100 $$

### Differential Scanning Calorimetric (DSC) Studies

The crystallinity of the CIL powder and the PCL and PCL + CIL electrospun fibers was determined by differential scanning calorimetry (DSC, Perkin Elmer DSC 8000) analysis. The calibration procedure was a multi-step process which involved baseline optimization, sample temperature calibration, furnace and the heat flow control. As reference, indium and lead were used for temperature and enthalpy calibration. All samples were analyzed in the temperature range from −30 to 190°C. The DSC runs for each sample were recorded in both heating and cooling modes at a temperature rate of 10 K/min. All experiments were performed under a nitrogen gas flow. The degree of crystallinity (Xc) was calculated using the following equation:

4 $$ \mathrm{Xc}\left(\%\right)=\frac{\left[\Delta  \mathrm{Hm}\right]}{\left[\Delta  {\mathrm{Hm}}_0\right]}\ast 100 $$

where, ∆Hm (J/g) is the enthalpy of melting derived from DSC curves, and ∆Hm_0_ (J/g) is the enthalpy of melting 100% crystalline polymer. For PCL, ∆Hm_0_ is 139.5 J/g (30).

### X-ray Diffraction (XRD) Studies

The structural properties of the fibers were analyzed by X-ray diffraction (XRD). A high-resolution Empyrean diffractometer (PANalytical) was used, employing CuKα_1_ radiation (1.54 Å) and operating in the Bragg-Brentano geometry. The 2 Theta scans were recorded in angles ranging from 5 to 50 (2θ) in continuous scan mode. The PCL crystallite size was calculated from the Scherrer equation:

5 $$ D=\frac{k\times \lambda }{FWHM\ast \cos \theta } $$

where D is crystallite size, k is the shape factor with a value of 0.94, *λ* is the X-ray wavelength, line broadening is marked as the full width at half maximum (FWHM) and θ is the diffraction angle.

### Fourier Transformed Infrared (FTIR) and Raman Spectra Analysis

The Fourier transformed infrared (FTIR) absorption spectra of fibrous PCL and PCL/CIL mats were recorded using Bruker Tensor 27 FTIR spectrometer with diamond ATR, in a spectral range of 400 cm^−1^ to 4000 cm^−1^ with a resolution of 4 cm^−1^. The background spectrum was run with an empty ATR cell without any sample. The background spectrum and sample spectra were measured at room temperature.

Raman spectra for all produced samples were obtained by using an inVia Renishaw confocal Raman microscope (Renishaw) with a 50×/0.75 microscope objective (LEICA) and by an engaging laser of 514 nm. Each Raman spectrum was obtained in the range of 1000 to 2000 cm^−1^ corrected by the WiRE™3.3 software connected to the instrument.

### Mechanical Properties Determination

The nanohardness and elastic modulus of the single fibers collected under 0 rpm were measured by nanoindentation (Hysitron TI 950 TriboIndenter) using a Berkovich diamond tip with a maximum penetration depth below 50 nm. Hardness and elastic modulus values were determined from the load-displacement curves by the Oliver–Pharr method (31). Samples were scanned using 0.75 μN and 0.1 Hz to avoid displacements during the topographic survey. A long drift correction of 300 s was used to account for both thermal and elastic drift, with typical drift values being 0.05 nm/s.

### ***In Vitro*** Release Studies

An *in vitro* release studies were performed using USP 4 apparatus. Operated in the closed configuration, the semiautomated SOTAX CE 7 smart apparatus (Basel, Switzerland) was linked to a piston pump SOTAX CP 7–35 (Basel, Switzerland) and a dual-beam UV-Vis spectrophotometer Evolution 300 PC (Thermo Scientific, Madison, WI/US) equipped with a linear 8-cell changer and set of quartz flow-through cells (path length of 12 mm; Starna Scientific, Hainault, UK) for a direct online analysis. For each formulation of electrospun materials 8 mm disks were cut from an electrospun sample and placed between two layers of glass beads in apparatus cells. Six standard cells with a diameter of 12 mm and one empty cell (used as reference), were inserted in a heating jackets and used per experiment. The temperature was set at 37°C. During the test 125 mL, 250 mL or 500 mL of deaerated phosphate buffered saline (PBS; pH = 7.4) was pumped from the reservoir through each cell with a flow rate of 16 mL/min maintained at ±2%. The media volume was adjusted to each formulation in order to achieve sink conditions for drug content in obtained structures. Deaeration of the medium was performed using SOTAX Media Preparation Station (Basel, Switzerland) in order to minimize the risk of air bubbles. *In vitro* studies were carried out for 48 h and the concentration of CIL was determined every 1 h by UV absorption spectrophotometer at 257 nm. Data acquisition and processing were performed with WinSOTAXplus Advanced Dissolution Software (Basel, Switzerland). Each experiment for analyzed formulations was carried out in quadruplicate.

The kinetics of CIL release from electrospun materials were determined using DDSolver software (32) by fitting obtained results to different kinetic models (33,34):

1. Ritger-Peppas model used to analyze release from polymeric drug dosage forms where the mechanism is not well known or more than one type of mechanisms might be involved

6 $$ \frac{M_t}{M_{\infty }}=k{t}^n $$

2) Simplified Higuchi model developed to study the release of water soluble and low soluble drugs incorporated to solid matrices

7 $$ \frac{M_t}{M_{\infty }}={k}_H{t}^{1/2} $$

3) Hixson-Crowell model assuming that the release rate is limited by the drug particles dissolution and not the diffusion through polymer matrix

8 $$ {M}_0^{1/3}-{M}_t^{1/3}={k}_{HC}t $$

4) Peppas-Sahlin model that includes both Fickian contribution and polymer relaxational contribution, associated with stresses in polymers in aqueous medium

9 $$ \frac{M_t}{M_{\infty }}={k}_1{t}^{0.5}+{k}_2t $$

In these models M_t_ is the cumulative amount of the active substance released at time t; M_∞_, M_0_ represents the starting amount of the active substance, n is the release exponent, whereas k_H_, k_HC_, k, k_1_, k_2_ are constants. The similarity factor of release profiles was also determined.

## Results and Discussion

### Electrospinning and Morphological Characterization

Polycaprolactone was chosen for the preparation of the drug-loaded fibers since it demonstrates good compatibility with various drugs and thus can be used both in drug delivery system and applied in tissue engineering (35). Analyzed polymer concentration (6% (wt), 8%(wt), 10%(wt)) were determined based on previous reports and our optimization process (7,36).

The water solubility of CIL at room temperature is 0.0324 mg/mL (37). As CIL is soluble in the mixture used to dissolve PCL (chloroform: methanol 3:1) (30), it was incorporated in the electrospun fibers through direct blending. In this technique, the active substance is suspended or dissolved in the polymer solution. In the preformulation stage of electrospun product development, it is crucial to take into account both polymer and active substance solubility in the organic solvents used to create polymer solution. Therefore, in our study, chloroform was the basic solvent in the organic solvent mixture, which was able to solubilize both PCL and CIL.

**Fig. 2:**
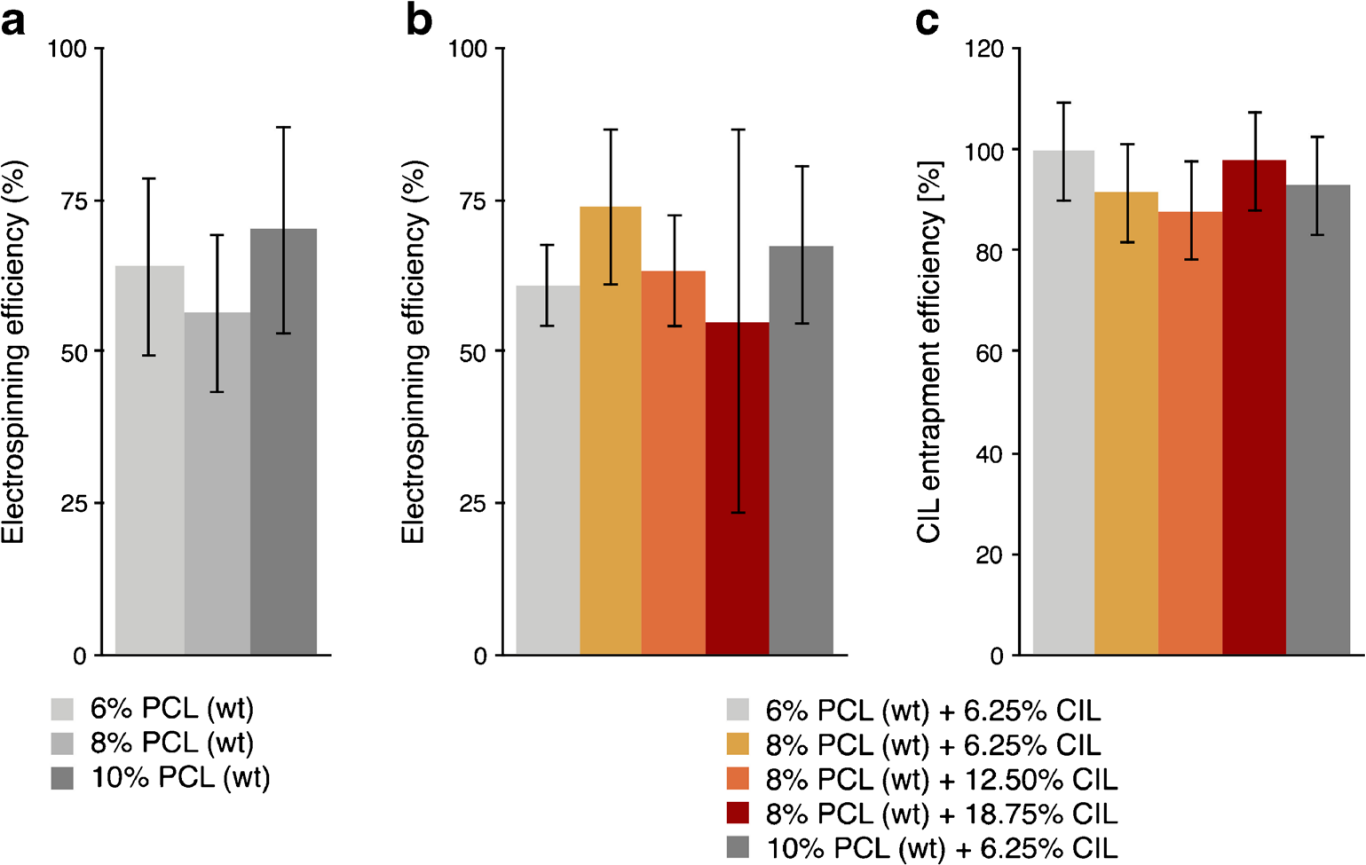
Calculated electrospinning efficiencies for PCL fibrous mats (**a**) and CIL loaded PCL fibrous mats (**b**) obtained by weighting substrates and products. Estimated CIL entrapment efficiencies (**c**) for various CIL loaded fibers formulations based on CIL absorbance value (at 257 nm) after dissolving electrospun tubular structures.

Additionally, methanol was added to the starting solution to reduce the evaporation rate of chloroform and enable uninterrupted fibers formation, thus preventing the polymer solution from clogging the spinneret (30,38). Analyzed CIL concentrations were 6.25%, 12.50%, 18.75% based on the weight of PCL. Presented loadings of CIL was selected in order to understand miscibility of both components and their interactions in the final product. Depending on the type of formulation and electrospinning process efficiency CIL loadings corresponded to approximately 5 mg to 18 mg of active substance per produced tubular structure. Figure 2 shows the calculated values of the process efficiency (Fig. 2 (a,b)) and entrapment efficiency (Fig. 2(c)). No significant differences in electrospinning efficiencies were observed between formulations of pure PCL and drug-loaded PCL fibers (Fig. 2 (a,b)).

As depicted in Fig. 2(c), high drug loading efficiency was achieved for various drug-loaded fibers formulations. However, it is possible that small quantities of the drug were trapped on the inner surface of the syringe, silicon tube or spinneret, which contributed to lower than 100% CIL entrapment efficiency for some formulations (39). Nevertheless, these inconsistencies were not significantly correlated with polymer concentration or CIL content used in selected formulations. SEM micrographs were analyzed to observe the effect of applied voltage, polymer solution composition and collector parameters on the morphology of electrospun fibers. The obtained results are presented in Table I and in electronic supplementary information in Table S1.

**Table I:** A Comparison of the Average Diameters and Alignment Coherencies of Fibers from Inner and Outer Layer of Electrospun PCL Tubular Structures Collected on a Rotating Collector and a Stationary Collector

Polymer solution in chloroform: methanol	Rotation speed (rpm)	Average diameter (μm) ± SD		Average coherency ± SD	
		inner	outer	inner	outer
8% PCL	0		0.81 ± 0.42	–	–
	1000	1.17 ± 0.47	1.18 ± 0.51	0.23 ± 0.11	0.35 ± 0.13
	2000	2.48 ± 0.95	2.33 ± 0.93	0.28 ± 0.13	0.56 ± 0.13
	4000	2.12 ± 0.68	2.48 ± 0.68	0.28 ± 0.12	0.61 ± 0.14
8% PCL +6.25% CIL	0		1.17 ± 0.20	–	–
	1000	1.38 ± 0.41	2.15 ± 0.57	0.33 ± 0.15	0.37 ± 0.17
	2000	1.38 ± 0.42	1.34 ± 0.34	0.25 ± 0.14	0.37 ± 0.17
	4000	0.77 ± 0.36	1.55 ± 0.53	0.21 ± 0.13	0.21 ± 0.11
8% PCL +12.50% CIL	0		1.67 ± 0.30	–	–
	1000	0.96 ± 0.53	1.24 ± 0.52	0.28 ± 0.12	0.27 ± 0.13
	2000	1.08 ± 0.38	1.76 ± 0.48	0.22 ± 0.10	0.23 ± 0.14
	4000	1.50 ± 0.78	1.43 ± 0.47	0.20 ± 0.11	0.24 ± 0.11
8% PCL +18.75% CIL	0		1.56 ± 0.41	–	–
	1000	1.68 ± 0.65	1.55 ± 0.43	0.15 ± 0.07	0.29 ± 0.10
	2000	1.15 ± 0.32	1.52 ± 0.43	0.16 ± 0.08	0.17 ± 0.08
	4000	1.51 ± 0.47	1.79 ± 0.51	0.15 ± 0.07	0.20 ± 0.10

Firstly, the optimal applied voltage was determined for electrospinning PCL solution 6% (wt), 8% (wt), 10% (wt) with and without CIL, on a non-rotating metallic plate acting as a collector. Polymer solutions containing PCL concentrations higher than 10% were eliminated at an early stage of the experiment as they resulted in either an unstable jet and/or a liquid whose viscosity was too great to allow it to easily pass through the spinneret (40). The PCL fibers were found to be easily produced at a range of voltages. The mean diameter of the CIL-loaded fibrous mats had a lower standard deviation than PCL fibers (Table I). The overall Fiji analysis of fiber diameter based on SEM micrographs revealed a distribution of sizes characteristic of electrospun PCL fibers (36). What should be mentioned is the fact that the data collected for the population of measured fibers is actually that of a single long fiber. As the electrospinning process is continuous due to a constant supply of the polymer solution, a single long thread of polymer is deposited on the collector. Therefore, the mean diameter is calculated based on the diameters of various segments of continuously deposited polymer fiber.

Tubular structures were obtained on a rotating drum collector with a programmed rotation speed. The results showed that average fiber diameter generally decreased as the polymer concentration increased from 6% (wt) to 10% (wt) on both the inner and outer surface of tubular structures, either with or without CIL (Table S1). In case of the fibers made of 6% (wt) PCL solution, the structures were characterized by bead formation which significantly increased the mean diameter (Table S1). The formation of these bead-like structures was caused by instability in the jet surface tension and its low viscoelasticity (41). These circumstances forced a liquid jet to assume a smaller surface area per unit mass thus leading to a sphere formation. Numbers of sphere and spindle-like beads (Fig. S1) were formed along very thin fiber between them. This specific morphology caused a significantly greater mean diameter of fibers and greater deviation of mean fiber diameter (Table S1).

**Fig. 3:**
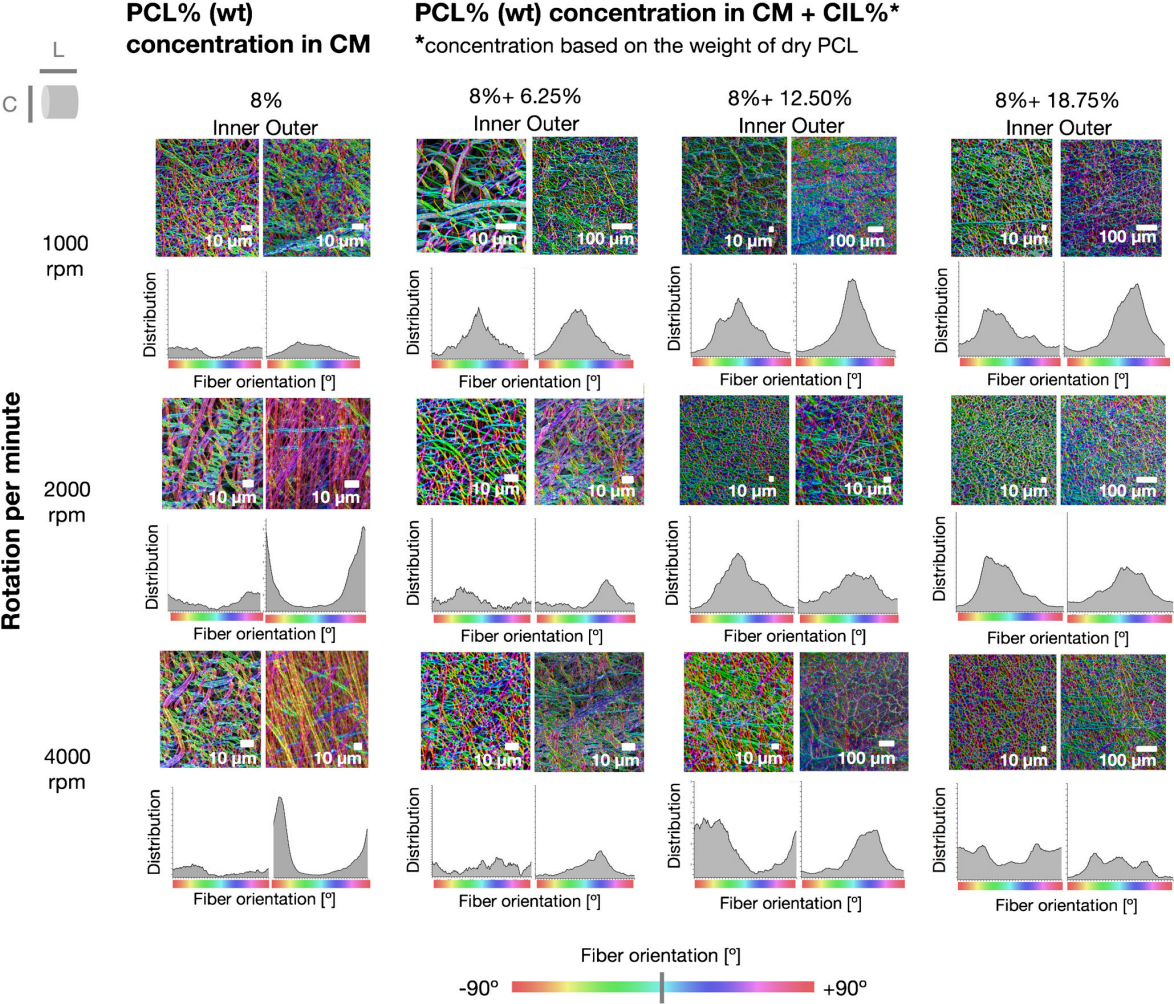
Fiber orientation presented as a color-coded SEM micrographs of various formulations of PCL fibers. Fiber orientation angle corresponds to a color from the bar presented at the bottom. Histograms demonstrating fiber orientation distribution are presented below each color-coded SEM micrograph. L and C refer to the longitudinal and circumferential directions of the electrospun sample.

Kim *et al*. report that the alignment and assembly of the fibers can be controlled by a programmed increase in the rotation speed of the drum collector (42). Figure 3 represents a graphical visualization of the fiber orientation in both the inner and outer surfaces of the electrospun tubular structures, with and without CIL, which had been prepared at different rotation speeds. Both graphical and numerical (Fig. 3, Table I, Fig. S2 in electronic supplementary information) results show that by changing the rotation speed of the collector from 1000 to 4000 rpm, the alignment and coherency coefficient of PCL fibers at the outer surface of the tubular structure was also increased. No such tendency was observed for the inner surface of the electrospun tubular structure, as fibers located on the inner side demonstrated smaller alignment and thus a lower coherency coefficient than the fibers on the outer side of the electrospun structure. A potential explanation for this difference in the observed coherency values is connected with process stabilization over time.

In the case of CIL-loaded PCL fibers, rotation speed was found to have a smaller influence on fiber alignment, as indicated by lower coherency coefficient values (Table I). The difference between the inner and outer surface of the electrospun structures was also less apparent.

**Fig. 4:**
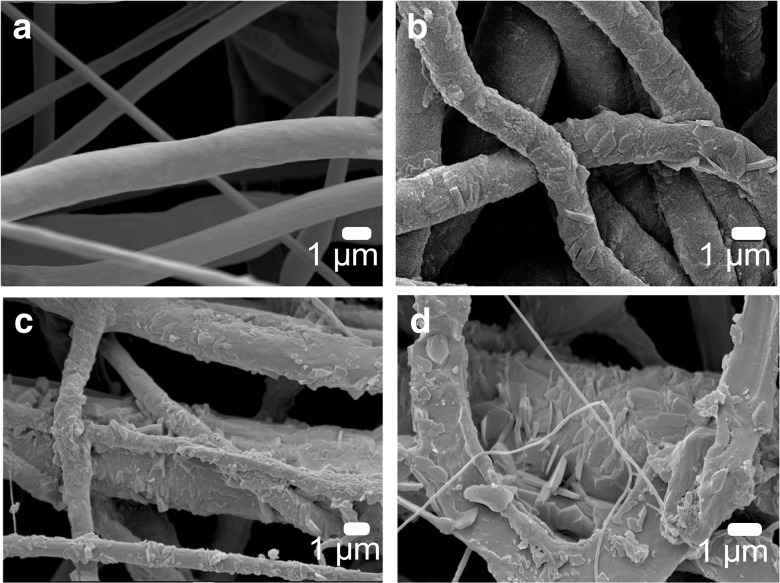
SEM micrographs of 8% (wt) PCL fibrous mats: without CIL (**a**), loaded with 6.25% CIL (**b**), loaded with 12.50% CIL (**c**), loaded with 18.75% CIL (**d**).

Drug loading is known to significantly influence the morphology of electrospun fibers (43) and this was also observed in the present study. Figure 4(a) shows SEM micrograph of PCL fibers without CIL loading. It demonstrates smooth surface of electrospun fibers made of pure PCL. SEM micrographs depicted visible CIL crystals even at the lowest CIL loading (Fig. 4(b)). The mean diameters of CIL crystals loaded into the PCL fibers spun from 8% polymer solutions were found to be 263 ± 217 nm, 414 ± 241 nm and 553 ± 362 nm for 6.25 (Fig. 4(b)), 12.50 (Fig. 4(c)) and 18.75 (Fig. 4(d)) % CIL loading respectively.

A previous study reported the crystallization of hydrophilic drug on the surface of electrospun fibers created by electrospinning with a hydrophobic polymer (44). Both the properties of the solvent and the drug-polymer interaction affected the electrospinning process, drug encapsulation, and the drug release kinetics (44). In this study, crystallization occurred despite the hydrophobic nature of both drug and polymer, which showed that hydrophobicity does not promote drug encapsulation in hydrophobic PCL fibers and is not responsible for outer surface crystallization. According to Seif *et al*. this could be explained by the high solubility of the drug in an organic solvent (45). The 3:1 *v*/v mixture of chloroform and methanol easily dissolved both PCL and CIL. CIL is freely soluble in chloroform and only slightly soluble in methanol. This difference in solubility potentially caused CIL molecules to leave with the fast-evaporating chloroform during the jet elongation phase of the electrospinning process when the evaporation of organic solvents occurs. This process eventually led to CIL crystallization on the outer surface of PCL fibers (Fig. 4(b-d)).

### Hygroscopicity

It is important for a medical material to have low hygroscopicity, especially in the case of pharmaceutical products because it will determine their storage conditions. The hygroscopicity was determined based on the pharmacopeial method (29). The percentage increase in mass was estimated to be 1.82 ± 1.61% for PCL fibers and 1.33 ± 0.26% for PCL fibers loaded with CIL. According to data provided by the European Pharmacopeia, the identified gain in mass fell in the range from 0.2 to 2% thus indicating that both electrospun materials were slightly hygroscopic. As the first step of PCL degradation is based on hydrolytic degradation, the hygroscopicity of electrospun PCL is a crucial issue. Amorphous regions of PCL fibers may absorb water, which acts as a medium for the degradation process; however, this water absorption process was found to be reversible (46).

### Evaluation of Drug Presence and Crystallinity

The drug loading of electrospun fibers through direct blending involved initial drug dissolution in the polymer solution which was followed by organic solvent evaporation and fiber solidification on the grounded collector. This process could lead to significant changes in the crystal structure of the incorporated drug. DSC, FT-IR, Raman spectroscopy and XRD studies were performed in order to confirm the presence of the drug in the electrospun fibers, and to evaluate the structural changes in the polymer matrix and drug crystallinity after the electrospinning process.

#### Differential Scanning Calorimetric (DSC) Studies

**Fig. 5:**
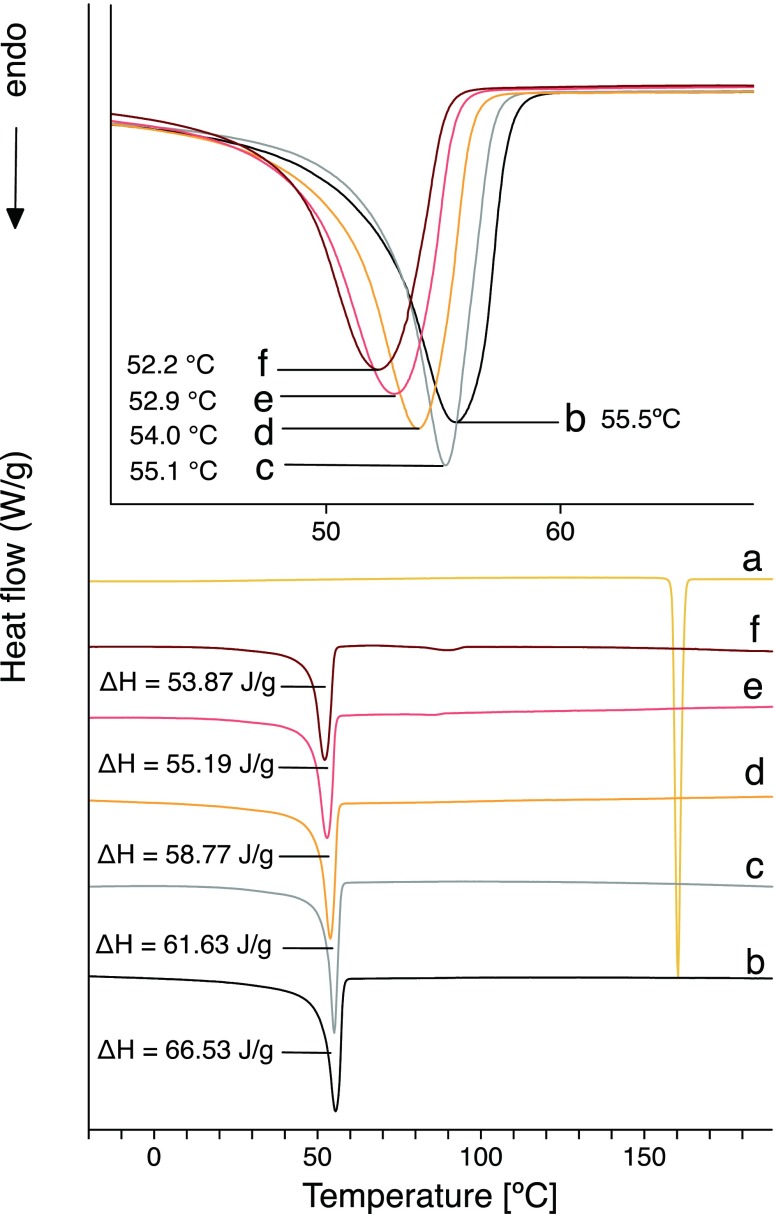
DSC curves of CIL powder (**a**), PCL pellets (**b**), PCL fibrous mats (**c**) and PCL fibrous mats with 6.25% (**d**); 12.50% (**e**); 18.75% (**f**) of CIL. Inset represents stacked thermographs of various fiber formulations, visualizing shifts in the melting temperature of PCL.

The DSC curves revealed endothermic peaks at 55.5°C for PCL and 160.3°C for cilostazol, (Fig. 5) (24,47). The melting temperature of cilostazol revealed its crystalline form. This was indicated as form A, which is the most thermodynamically-stable polymorph but the least soluble in an aqueous environment (24).

A characteristic endothermic melting peak for PCL was found in the thermographs of both the PCL fibers and PCL fibers loaded with various concentrations of CIL, which indicated that obtained PCL fibers had a crystalline structure. Interestingly, the thermographs for the CIL-loaded fibers showed only a peak for the presence of PCL. The lack of any detectable crystalline domain for CIL within the electrospun fibers indicated that the drug was molecularly dispersed within the polymer matrix or was present within the fibers in an amorphous state. However, SEM micrographs demonstrated that at least some part of CIL load was present on the surface of fibers and DSC study showed no glass transition during the analysis of CIL-loaded fibers. Still, it could not be excluded that some part of CIL was dispersed within PCL matrix of the fiber or that the crystalline part of CIL present within fibers was below the detection limit for DSC analysis. However, in case of latter, a more significant portion of amorphous CIL would mean that characteristic signals or peak shifts for amorphous CIL would be visible in other techniques. Our study found no characteristic features of other polymorphic or amorphous forms of CIL in FTIR, Raman or XRD spectra. Additionally, differences in the melting temperature of PCL and CIL could partially account for the lack of any peak for CIL on the thermograph. As PCL has a lower melting temperature than CIL, the electrospun PCL fibrous mat melted first, with the CIL nanocrystals depicted on the SEM micrographs dissolving in the melted PCL (48).

The characteristic PCL peak for CIL-loaded fibers was shifted to a lower melting temperature than that observed for PCL fibrous mats, suggesting that the presence of CIL influences the crystalline structure of PCL. This reduction in the overall crystallinity of electrospun PCL when compared to PCL, could be explained by the processes which happen during the elongation phase in the electrospinning process. In this phase, polymer chains are elongated along the electrostatic field, while rapid organic solvent evaporation occurs. This leads to fast polymer solidification, which decreases the molecular order and overall crystallinity of the substance (49).

The observed shift in the melting temperature of CIL loaded PCL fibers was correlated with an increase in CIL content. The enthalpy decreased for 6.25%; 12.50%; 18.75% CIL loaded PCL fibers to the value of Δ*H = 58.77 J/g; ΔH = 55.19 J/g; ΔH = 53.87 J/g,* respectively; in comparison, the enthalpy value for PCL fibers was Δ*H = 61.63 J/g.* The decrease in enthalpy value was also correlated with an increase in CIL content. Based on these obtained enthalpy values, the degree of crystallinity (Xc) was determined for the samples: Xc was found to be 44.18% for PCL fibers, and 42.13%; 39.56% and 38.62%, respectively, for the 6.25%, 12.50% and 18.75% CIL-loaded PCL fibers. The amorphous region of the polymer fibers was estimated to be around 60%, which corresponded well with data reported elsewhere (50).

DSC analysis was also carried out for PCL and PCL drug-loaded fibrous mats electrospun under different collector rotation speeds (see electronic supplementary information Fig. S3). It was found that the rotation speed had no effect on the melting point of PCL. The increase in collector rotation speed from 1000 to 4000 rpm was found to have no effect on enthalpy values and the crystallinity of the electrospun fibers. However, other reports have found that greater collector rotation speeds result in reduced crystallinity of electrospun PCL fibers (51).

DSC analysis showed that the polymer was not changed significantly during electrospinning process and the drug was successfully incorporated into the electrospun fibers.

#### Fourier Transformed Infrared (FTIR) Spectra Analysis

**Fig. 6:**
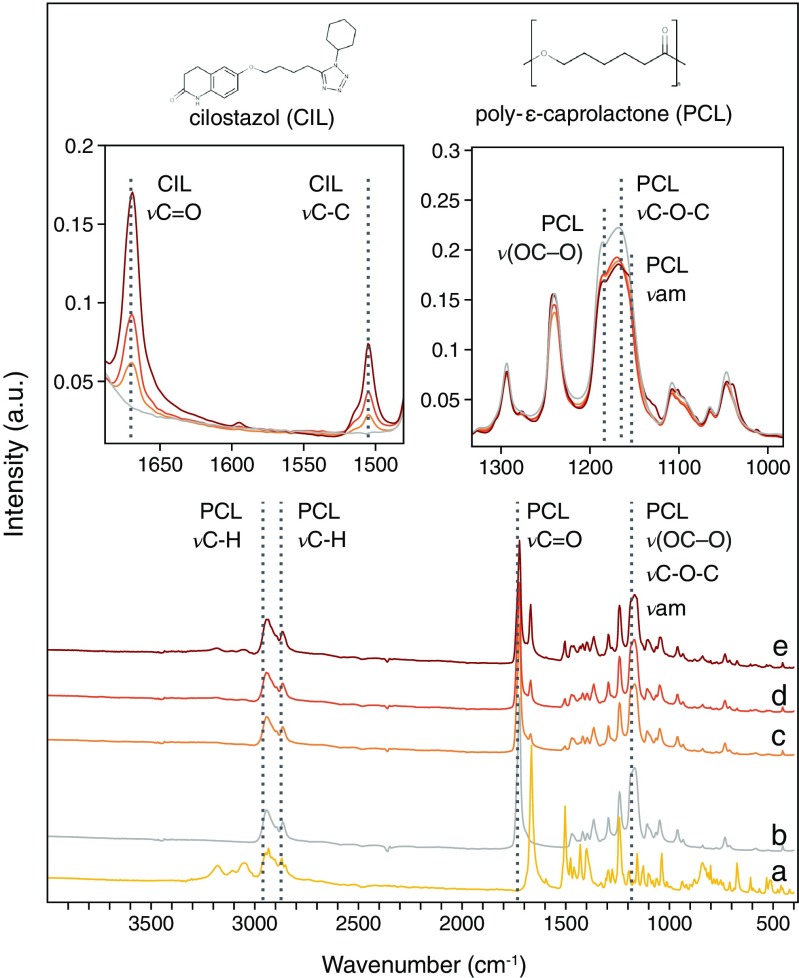
FTIR spectra of CIL powder (**a**), as prepared PCL fibrous mats (**b**) and PCL fibrous mats with 6.25% (**c**); 12.50% (**d**); 18.75% (**e**) of CIL. The left inset represents stacked spectra of various fiber formulations: the intensity of the peaks increases with increasing CIL content. The right inset demonstrates lower peaks intensity, which is characteristic for PCL with the presence of CIL.

When developing electrospun DDS, it is crucial for the polymer and active substance to be compatible. The FTIR spectra of CIL (Fig. 6(a)), PCL (Fig. 6(b)) and CIL-loaded fibrous mats (Fig. 6(c-e)) were obtained. The PCL peaks at 2945 cm^−1^ and 2864 cm^−1^ indicate asymmetric and symmetric CH_2_ stretching (52). The characteristic peak of PCL fibrous mats at 1720 cm^−1^ was related to carbonyl stretching (Fig. 6(b)). The broad band in the region 1150 cm^−1^ – 1200 cm^−1^ consists of three overlapping peaks: A peak at 1187 cm^−1^, which could be referred to OC–O stretching, 1168 cm^−1^ to symmetrical COC stretching, and 1155 cm^−1^ to C–O and C–C stretching in the amorphous phase (52).

Cilostazol (Fig. 6(a)) showed a peak at 1505 cm^−1^ related to aromatic C-C stretching, another at 1667 cm^−1^ corresponding to C=O stretching and a group of peaks at 3312 cm^−1^ to 2807 cm^−1^ corresponding to aromatic and aliphatic CH stretching.

The intensity of the peaks at 1505 cm^−1^, related to aromatic C-C stretching, and at 1669 cm^−1^ assigned to C=O stretching, was found to increase with increasing CIL concentration, as shown in the left inset in Fig. 6. These phenomena were not observed in PCL fibers. Additionally, some of the peaks between 3312 cm^−1^ and 3000 cm^−1^ were detected only in fibrous mats with higher CIL content (18.75%; Fig. 6(e)). No significant shifts in CIL wave numbers were detected for the analyzed PCL fibers loaded with CIL. The peaks at 1669 cm^−1^ and 1505 cm^−1^ were characteristic for form A of CIL. In comparison, these peaks are shifted to 1662 cm^−1^ and 1504 cm^−1^ for form B and to 1674 cm^−1^ and 1504 cm^−1^ for form C (53,54).

All obtained spectra for PCL fibrous mats and PCL drug-loaded fibrous mats contained all the characteristic peaks for the polymer and the incorporated drug. This indicated that drug-polymer interactions were weak after the electrospinning process. Such interactions as hydrophobic interaction, hydrogen bonding and electrostatic forces could improve drug-polymer compatibility, and these would be demonstrated on FTIR spectra by the presence or disappearance of certain peaks (24,55). The interaction between cilostazol and PCL could occur *via* hydrogen bond formation. The N-H moiety in cilostazol molecule is capable of forming hydrogen bonds with the C-O-C moiety in PCL. The evidence provided by FTIR spectra showed no visible peak shifts in the spectra of drug-loaded fibrous mats (Fig. 6). However, the intensities of peaks at 1187 cm^−1^, 1168 cm^−1^ and 1155 cm^−1^ referred to OC–O stretching, symmetrical COC stretching, C-O and C-C stretching were reduced and it was not proportional to cilostazol content (Fig. 6 right inset). The reduced intensities of those peaks could indicate that OC-O and C-O stretching of PCL monomers were prevented, which may be caused by hydrogen bond formation between PCL and CIL. Additionally, it could also indicate changes in PCL crystallinity after CIL addition, which is consistent with DSC studies (Fig. 5).

The FTIR spectra confirmed the presence of CIL in the electrospun fibrous mats and demonstrated that the CIL encapsulation by blend electrospinning is a facile method to obtain drug/polymer composite fibers.

#### Raman Spectra Studies

**Fig. 7:**
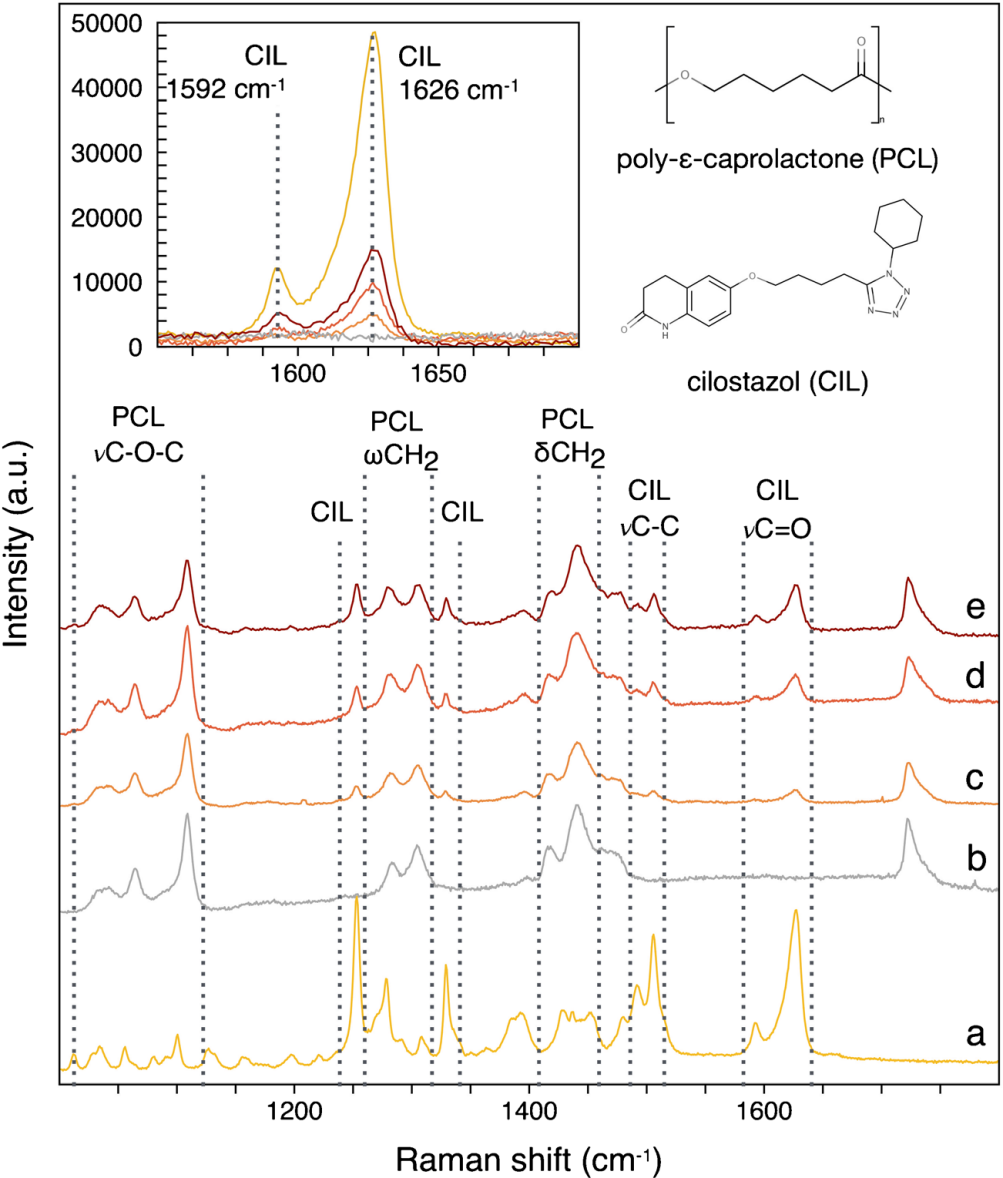
Raman spectra of CIL (**a**), PCL fibrous mats (**b**) and PCL fibrous mats with 6.25% (**c**); 12.50% (**d**); 18.75% (**e**) of CIL. The inset represents two characteristic peaks for form A of CIL which intensity increased with an increase in drug content.

Figure 7 represents the Raman spectra of CIL powder (Fig. 7(a)), PCL (Fig. 7(b)) and PCL drug-loaded fibrous mats (Fig. 7c-e) in the region from 1000 to 1800 cm^−1^. PCL is a semicrystalline and hydrophobic polymer (56,57). For PCL fibrous mats (Fig. 6(b)), several narrow peaks were observed including one in the range of 1033–1107 cm^−1^, assigned to skeletal stretching, 1282–1304 cm^−1^, related to ω CH_2_, and 1415–1470 cm^−1^, corresponding to δ CH_2_. Moreover, a narrow peak was observed at 1720 cm^−1^ which was related to the ν C=O stretching mode. No shift in peaks position was observed.

Raman spectra of PCL fibrous mats loaded with CIL (Fig. 7(c-e)) showed peaks characteristic for CIL. The Raman peak at 1626 cm^−1^ corresponded to ν C=O whereas the peak at 1505 cm^−1^ demonstrated aromatic chain stretching for form A. The band at 1592 cm^−1^ was present on the spectra of all analyzed formulations of drug-loaded PCL fibrous mats (Fig. 7(c-e)) and is a characteristic peak for form A of CIL. According to the literature, the peak at 1592 cm^−1^ is absent in the Raman spectra of form B (9). The peaks at 1253 cm^−1^ and 1329 cm^−1^ noted on the spectra of CIL-loaded fibrous mats (Fig. 7) correspond to form A of CIL. According to Stowell and Whittle, the peaks at 1253 cm^−1^ and 1329 cm^−1^ of form A are shifted to 1246 cm^−1^ and 1334 cm^−1^ for form B, and to 1255 cm^−1^ and 1324 cm^−1^ for form C (24,55). As various analytical techniques are necessary for active substances identification and solid-state excipients characterization, DSC and Raman and FTIR spectroscopies should be regarded as complementary methods. Moreover, together they provide data necessary for the evaluation of crystal structure and polymorphism of excipients used in the formulation, which is an important part of preformulation of a newly developed drug delivery system. In case of CIL, the difference between polymorphic forms is connected with their thermodynamic stability and solubility in aqueous media. As-received CIL is in form A which is thermodynamically the most physically stable polymorph. Form B is the least stable polymorph but with the highest solubility in water. Properties of form C are located between those of form A and B. According to the heat of fusion rule, form B may spontaneously change to form A (24). This would change the solubility of CIL in aqueous media. CIL is classified as BCS class II drug, thus this change would significantly affect the release kinetics from polymer system. Based on data obtained from FTIR and Raman studies, it was confirmed that CIL retained its crystalline A form structure after the electrospinning process.

#### X-ray Diffraction (XRD) Studies

**Fig. 8:**
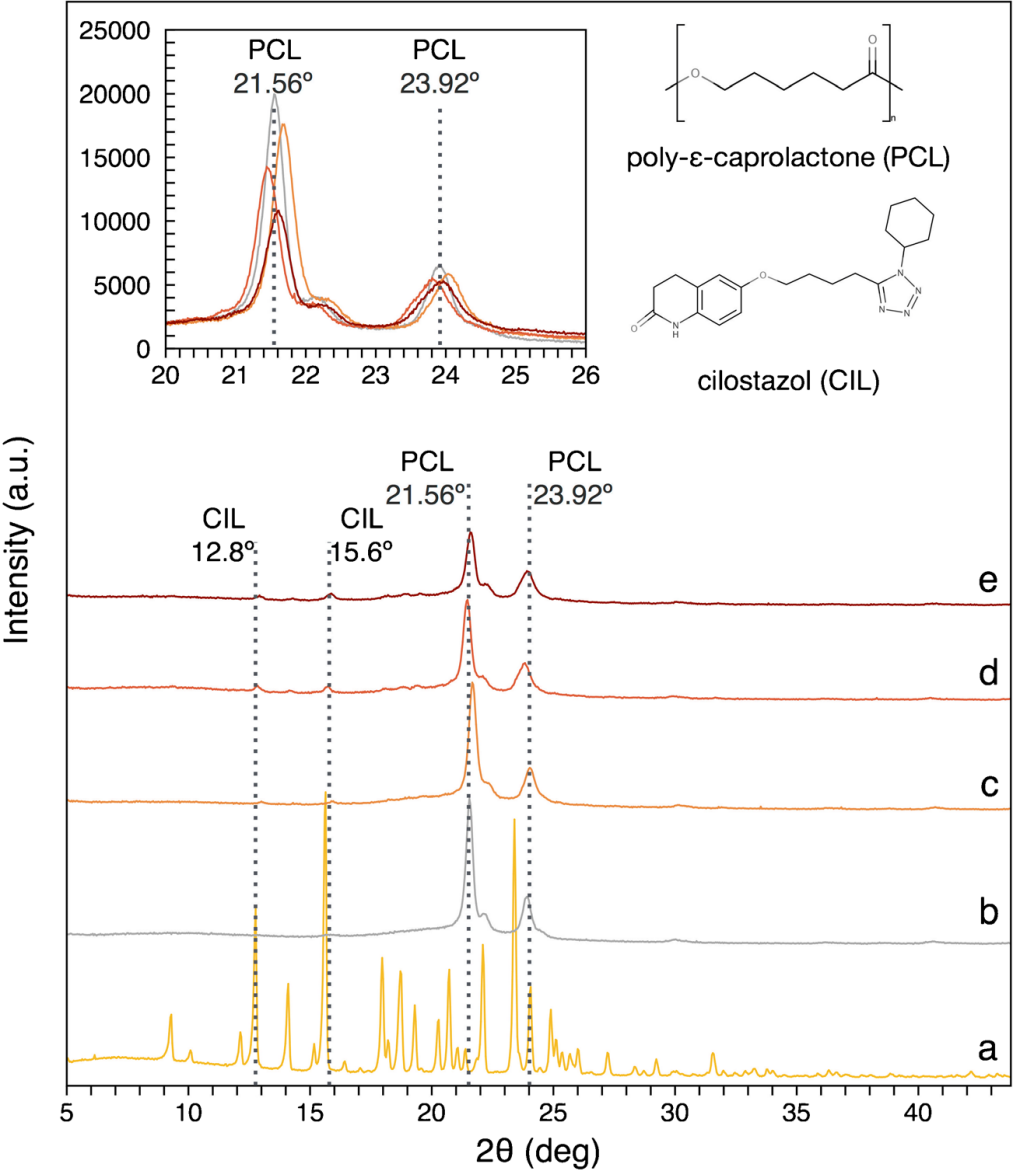
XRD patterns of the prepared PCL fibrous mats (**a**) and CIL powder (**b**) as well as PCL fibrous mats with 6.25% (**c**), 12.50% (**d**), 18.75% (**e**) of CIL. The inset represents stacked spectra of various fiber formulations and demonstrates the influence of CIL presence on the crystallinity of PCL.

XRD analysis was implemented to determine the crystallinity of the electrospun fibers. Figure 8 shows the XRD pattern for PCL fibrous mats (Fig. 8(b)) and PCL fibrous mats loaded with a various amount of CIL (Fig. 8(c-e)). Figure 8a shows the XRD pattern for CIL powder.

The X-ray diffraction pattern of PCL electrospun mats showed two major peaks at 2θ = 21.56° and 2θ = 23.92°, which are characteristic for electrospun PCL mats (58,59). However, minor differences in the positions of XRD peaks were observed between mats with different CIL content (Fig. 8 inset). According to Chew *et al*. they were caused by subtle changes in X-ray transparency which depended on the fiber diffraction and air scattering of photons (60).

The intensity of the 2θ = 21.56° peak decreased as the CIL content of the PCL fibers increased (Fig. 8(c-e)), indicating reduced PCL crystallinity of the electrospun fibers (60). Additionally, the greater width of CIL-loaded PCL fibrous mats peaks (2θ = 21.56°, 2θ = 23.92°) also suggested a decrease in the crystal size of drug-loaded electrospun mats when compared to pure PCL fibrous mats.

XRD patterns of fibers loaded with CIL (Fig. 8(c-e)) showed peaks near 12.8° and 15.6° as well as some minor peaks at 18.0°, 18.7° and 19.3° characteristic of form A of CIL (22,24,55).

XRD analysis was also performed for PCL and PCL drug-loaded fibrous mats with regard to the electrospinning collector rotation speed (see electronic supplementary information Fig. S4). For PCL drug-loaded fibrous mats, the intensity of the crystalline peaks was lower at higher rotation speeds of the drum collector. Nevertheless, DSC analysis of the same samples did not reveal any correlation between increase in rotation speed and decrease in crystallinity of electrospun PCL. In the case of the PCL fibrous mats, no particular pattern correlated with the increase in collector rotation speeds from 1000 to 4000 rpm was found. Moreover, our findings indicated that the observed decrease of crystallinity for drug-loaded fibers was connected with changes in surface roughness. The mats with incorporated CIL were characterized by a rough surface, which influenced the X-ray diffraction pattern (60).

The results of the XRD and DSC studies found that collector speed has no influence on the crystallinity of electrospun PCL within the collector rotation speed range from 1000 rpm to 4000 rpm. However, another study using a similar solvents mixture found that the crystallinity varies within the range from 0 rpm to 6000 rpm (51).

### Nanomechanical Properties

Cell growth is guided by a number of biophysical and biochemical cues present in the extracellular matrix (ECM) that regulate tissue restoration. ECM biochemical composition is complex and includes proteoglycans, glycosaminoglycan and proteins (collagen, elastin, fibronectin), which contain adhesive binding sites for cells thus guiding cell spreading and migration. The high molecular weight proteins are typically organized into a fibrillary network that provides substantial biophysical support by sufficient tensile strength and elastic modulus (61). During tissue restoration cells are guided by chemotactic substances including growth factors or cues present within ECM fibrils. However, cell movement requires substantial mechanical interaction with ECM. In order to restore tissue, their function is at least in part regulated by the mechanical equilibrium of local microenvironment, and in particular by the stiffness (Young modulus) of the surrounding ECM (62). Mechanical studies of PCL-based vascular grafts were reported in terms of compressive mechanical properties like Young’s modulus, ultimate tensile strength and strain or uniaxial tensile (7). However, nanomechanical properties are rarely reported. For a vascular graft made of electrospun fibers, designed to be used as an artificial cytoskeleton, it is important to consider its nanomechanical properties, as these will have important implications for cell function. Nanoindentation is a valuable method for evaluating the mechanical properties of nanostructures, as well as biological structures, like the extracellular matrix. Therefore, nanoindentation tests were conducted in order to analyze the effects of polymer concentration and active pharmaceutical ingredient addition on Young’s modulus (*E*) and hardness (*H*) of both PCL fibers and drug-loaded PCL fibers.

**Fig. 9:**
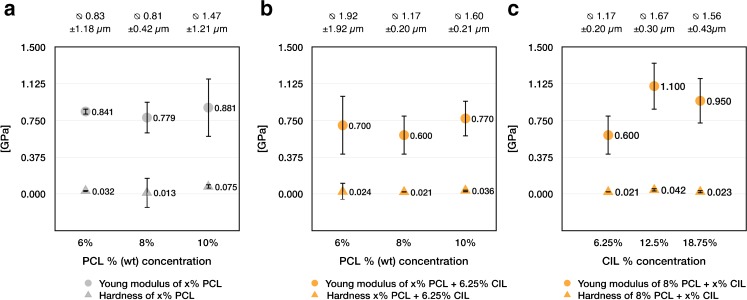
Mean fiber diameter, Young’s modulus and hardness of - PCL electrospun fibers as a function of PCL concentration (**a**), − PCL electrospun fibers with 6.25% of CIL as a function of PCL concentration (**b**), − 8% PCL electrospun fibers with CIL as a function of CIL concentration (**c**).

Average Young’s modulus and hardness values for fibers electrospun from 6% (wt) PCL solutions with or without CIL addition (Fig. 9(a-b)), were affected by bead-like deformations found along fibers and depicted by SEM micrographs (Fig. S1).

There were no significant changes in the Young modulus and hardness of PCL fibers, despite different PCL concentration in starting polymer solution (Fig. 9a). No significant changes in mechanical properties of produced fibers were observed after addition of 6.25% CIL to formulations with different concentrations of PCL in the starting solution (Fig. 9(a-b)). Small amounts of CIL (up to 6.25%) did not significantly change the mechanical properties of produced fibers. Nevertheless, the elastic modulus values obtained for PCL fibers were consistent with those found in the literature (63,64).

The rate of polymer solidification and crystallization is affected by solvent, polymer concentration and drug incorporation (60). To study the influence of drug loading on the mechanical properties of the fibers, a range of samples with different concentrations of CIL were also produced (Fig. 9(c)).

In our study, 8% PCL formulation + 6.25% CIL was characterized by a slight decrease in Young’s modulus (Fig. 9(c)) with a concomitant increase in fiber diameter (Fig. 9(c)) when compared to 8% PCL formulation without CIL (Fig. 9(a)). Fibers produced from 8% PCL solution with loading levels of 12.50% and 18.75% of CIL resulted in a greater mean fiber diameter, as well as in slight increase in Young’s modulus (Fig. 9(c)). However, this increase in Young’s modulus could not be solely caused by greater fiber diameter, as Fig. 9(b) demonstrates that even more notable change in fiber diameter did not change Young modulus in a significant manner. Nevertheless, as the size of the fiber has an influence on its mechanical properties, the exact nature of diameter effect remains unclear.

The changes in mechanical properties can be attributed to electrostatic interactions between fiber components (65) and crystalline structure of various fiber formulations (60,66).

The polymer–drug interactions are mainly caused by hydrogen bond formation and/or hydrophobic interactions between functional groups of both ingredients (67). Theoretically, electrostatic interactions in a form of hydrogen bonds between PCL and CIL are possible, as hydrogen donor and acceptors are present within PCL and CIL molecules. FTIR analysis did not reveal any second peak to PCL carbonyl stretching (Fig. 6) which would indicate hydrogen bonded carbonyl vibration. Moreover, CIL demonstrates carbonyl vibration within region ranging from 1650 to 1700 cm^−1^ which overlaps with potential new band location (Fig. 6). Reduced intensity of peaks in the region 1150 cm^−1^ – 1200 cm^−1^ could indicate that OC-O and C-O stretching of PCL monomers were prevented, which may be caused by hydrogen bond formation between PCL and CIL. However, in solid dispersion hydrogen bonds between polymer and drug tend to increase nucleation energy thus limiting crystal growth (67). Still, despite the possibility of hydrogen bond formation, no signs of limited CIL crystal growth was observed in our study (Fig. 4). Nevertheless, potential hydrogen bonds between PCL and CIL could influence nucleation process and to some extent mechanical properties.

It was also found that the addition of the drug and the fast evaporation of organic solvent during the electrospinning process could result in a reduction in overall crystallinity and thus induce the formation of a larger amorphous part of the fiber (60). However, as the results of the DSC studies (Fig. 5) indicate the differences in crystallinity between different types of drug-loaded fibers were quite small, it is, hence unlikely that the observed difference in crystallinity could fully account for the large difference in mechanical properties of electrospun fibers.

SEM micrographs (Fig. 4) revealed that surface crystallization of CIL increased with a greater drug content of electrospun fibers. As the nanoindentation is a surface survey, therefore, greater Young’s modulus of the fibers loaded with 12.50% and 18.75% CIL could be a result of the presence of CIL nanocrystals at the surface of the fibers. PCL and CIL also act as a composite system, where polymer chain movements are restricted by CIL nanocrystals, which reinforce the structure of drug-loaded electrospun fibers (60).

From the application perspective, our results match the nanomechanical properties of the collagen fibrils present in blood vessels (68). Furthermore, more rigid materials tend to promote cell spreading by withstanding the cellular tension during the proliferation phase of tissue regeneration (66). As fibers are the basic element on which the entire tubular structure relies on, it is useful to understand their mechanical properties. Especially, when their stiffness described by Young modulus value impacts cell movement along ECM (66).

### ***In Vitro*** Release Studies

**Fig. 10:**
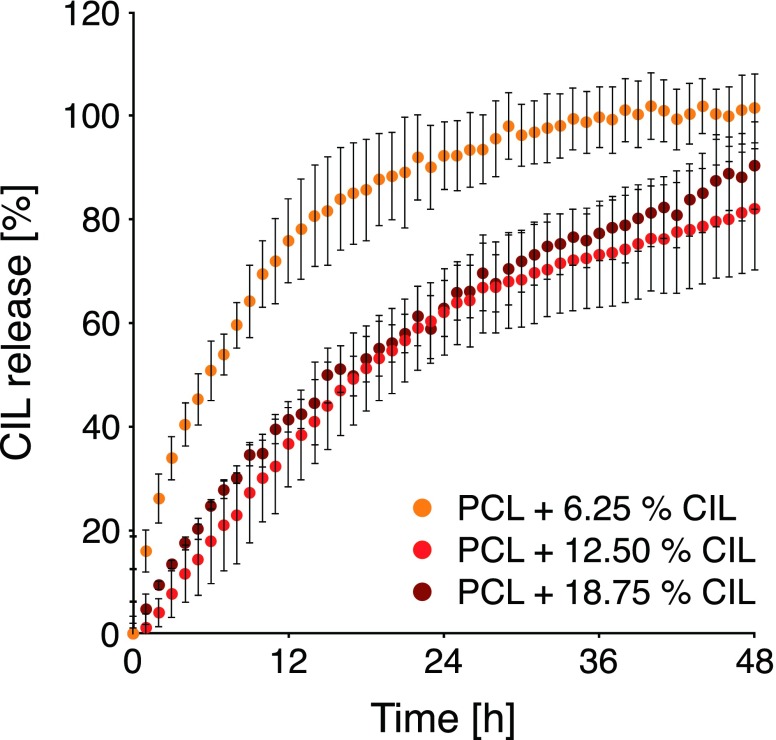
CIL release profiles from electrospun PCL materials loaded with 6.25%, 12.50% and 18.75% CIL (*n* = 4) in PBS (pH = 7.4) with temperature set at 37°C.

**Fig. 11:**
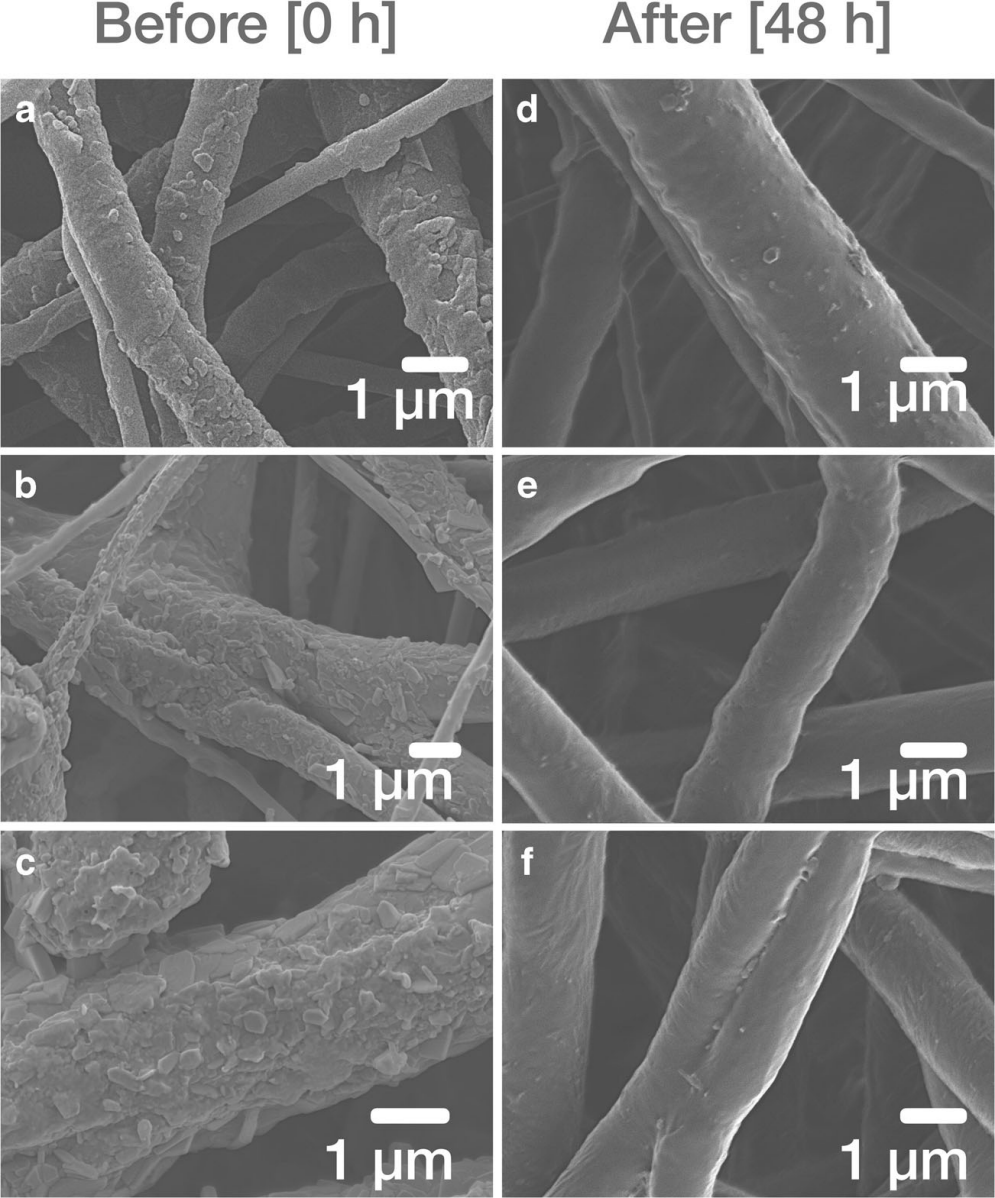
SEM micrographs before (**a,b,c**) and after (**d**, **e**, **f**) release studies of PCL materials loaded with 6.25% (**a**, **d**), 12.50% (**b**, **e**) and 18.75% (**c**, **f**) CIL.

The release kinetics of electrospun PCL materials containing various CIL loadings were determined *in vitro* and the effect of CIL loading on release profile was examined. The cumulative release profiles of CIL from the electrospun materials as the released fraction with respect to the total observed drug content within the sample are shown in Fig. 10. The times at which approximately 50% of the drug was released from 6.25%, 12.50% and 18.75% CIL loaded PCL electrospun structures were ca. 6.0, 17.5 and 16.8 h, respectively. Total release (101.52%) was obtained for PCL + 6.25% CIL formulation after 48 h. Additionally, the release profile reached the plateau in an investigated period. In case of PCL + 12.50% CIL and PCL + 18.75% CIL elution of 82.05% and 90.39% were noted, respectively. The release profiles revealed that PCL + 6.25% CIL formulation released CIL faster than formulation with higher CIL loading. The *in vitro* release profiles for PCL + 6.25% CIL were in line with the literature data reporting initial fast release phase of the incorporated drug followed by a slow release phase from the electrospun material (48). SEM micrographs (Fig. 11) revealed that electrospun PCL fibers retained its structure and integrity after dissolution testing with no signs of deformations, however CIL crystals at the surface of fibers were almost entirely eluted.

The similarity factor (f_2_) for obtained drug release profiles were determined. If determined f_2_ ∈[50,100] than a similarity of release profiles should be accepted. The drug release profiles obtained for fibers loaded with different amounts of CIL were significantly different, especially between PCL + 6.25% CIL and PCL + 12.50% CIL (f_2_ = 26.40), PCL + 18.75% CIL (f_2_ = 29.51) formulations. Release profiles of PCL + 12.50% CIL and PCL + 18.75% CIL were similar (f_2_ = 65.38). Surprisingly, increasing the CIL content from 6.25% to 12.50% resulted in a decrease in release rate and prolonged release duration. A similar tendency was found for other BCS class II drug (69). However, a study involving CIL dispersion within PLGA matrix reported that increasing the CIL content resulted in both an acceleration of the CIL release rate and a shortening of the release duration (23). These results prove that polymer type and drug-polymer ratio are significant formulation parameters that can be used to modulate drug delivery time scale.

Macroscopically electrospun materials can be regarded as thin polymer films. However, microscopically electrospun materials were composed of micro-sized fibers randomly arranged, which form a three-dimensional structure with voids between fibers. This specific architecture could affect the mechanism of release and the release rate. Fiber diameter could influence the release rate as it may change aqueous medium penetration and polymer swelling behavior. However various studies showed differing effects of fiber diameter on the release rate (70). In our study, the difference in fiber diameter, and therefore in the porosity of whole structure, were not significant (Table I).

The fiber surface morphology was significantly (Fig. 4(b-d)) altered by higher CIL loadings. SEM micrographs showed that CIL is located close to the surface of the fibers. Despite greater fiber surface coverage with CIL crystals in 12.50% and 18.75% CIL formulations, the release rate was significantly slower for these formulations. Differences in the average diameter of CIL crystal formations for various PCL + CIL formulations were noted. As CIL is a highly hydrophobic molecule classified as BCS class II substance, with low solubility in water, the drug crystal size difference could influence CIL dissolution into the aqueous medium. Dissolution of the drug could determine the rate of CIL release from electrospun materials. However, dissolution is not often reported as the rate-determining process in PCL based hydrophobic drug carriers.

Additionally, the appearance of highly hydrophobic CIL crystals on the surface of fibers could inhibit water absorption due to increased hydrophobicity of fibers surface.

The observed differences in the release rates of CIL could be also attributed to the crystalline microstructure of PCL. The drug release from electrospun semi-crystalline polymer takes place initially from its amorphous regions through water transport into the disordered matrix (71).

According to DSC data, increased CIL loading brought a small decrease of PCL fibers crystallinity. Therefore, it is possible that some part of CIL accumulated in the amorphous regions of PCL fibers, which caused the most of CIL dose to be released within 48 h.

The release profile may also be affected by the location of the active substance inside the polymer matrix (72). Based on FWHM of the PCL peaks found in XRD spectra, PCL crystallite size was calculated by Scherrer equation. For PCL, PCL + 6.25% CIL, PCL + 12.50% CIL, PCL + 18.75% CIL the crystallite size was 25 nm, 24 nm, 21 nm and 21 nm, respectively. It was found that CIL addition caused a decrease in PCL crystallite size. The most significant decrease in crystallite size was noticed between 6.25% CIL and 12.50% CIL loading. These results, suggest that PCL lamellae become smaller with increasing CIL content in the fibers, indicating CIL distortion of crystal regions of PCL fibers. Since the decrease was more pronounced for 12.50% and 18.75% CIL loadings it demonstrated that with higher CIL loadings greater portion of CIL was located in the crystalline domains of PCL matrix. Diffusion of the active substance is faster from amorphous regions of polymer matrix due to the higher mobility of polymer chains due to their disarrangement. As a result, aqueous medium penetrates polymer matrix more easily, leading to a faster release. Crystalline regions are more resistant to water penetration and hence might retard active substance diffusion.

To establish CIL release mechanism from electrospun PCL structures obtained *in vitro* release studies data were fitted to Ritger-Peppas, Higuchi, Hixson-Crowell, and Peppas-Sahlin model and results are presented in Table II.

**Table II:** Parameters Obtained by Fitting Four Different Models to the *in vitro* Release Data for Drug Release Kinetics Determination

Formulation	Higuchi			Hixson-Crowell			Ritger-Peppas					Peppas-Sahlin				
	*R* ^*2*^	*k*_*H*_	*k*_*H*_ SD	*R* ^*2*^	*k*_*HC*_	*k*_*HC*_ *SD*	*R* ^*2*^	*n*	*n* *SD*	*k*	*k SD*	*R* ^*2*^	*k*_*1*_	*k*_*1*_ *SD*	*k*_*2*_	*k*_*2*_ *SD*
PCL + 6.25% CIL	0.812	2.236	0.150	0.942	0.000	0.000	0.996	0.602	0.073	1.582	0.743	0.991	2.070	0.560	0.030	0.020
PCL + 12.50% CIL	0.940	1.535	0.229	0.975	0.000	0.000	0.991	0.875	0.056	0.130	0.068	0.989	0.304	0.216	0.045	0.007
PCL + 18.75% CIL	0.973	1.641	0.125	0.972	0.000	0.000	0.986	0.695	0.056	0.430	0.153	0.983	0.875	0.139	0.022	0.007

Considering the correlation coefficient values as obtained from different kinetic equations, the CIL release was found to follow Ritger-Peppas (R^2^= >0.986) and Peppas-Sahlin (R^2^= >0.983) models. Both models were applied for the first 60% of the measured fractional release. The release exponent (n) value of Ritger-Peppas model is an empirical parameter characterizing the release mechanism, which is valid up to 60% of cumulative drug release (33,34). In this study, the release exponent (n) values for PCL + CIL formulations were within the range typical for non-Fickian (anomalous) drug release mechanism (Table II). In anomalous transport, time scales of both diffusion and macromolecular relaxation of polymer chains are similar and both control the release mechanism. The Ritger-Peppas rate constant was sensitive to drug-polymer ratio in the examined 60% of total release, decreasing from 1.582 (R^2^ = 0.996) for PCL + 6.25% CIL to 0.130 (R^2^ = 0.991) and 0.430 (R^2^ = 0.986) for PCL + 12.50% CIL and + 18.75% CIL, respectively.

The Peppas-Sahlin model predicts that the diffusion mechanism in polymer matrices are due to two processes; diffusion from the polymer and polymer matrix relaxation (34,73). In this model k_1_ was the Fickian contribution coefficient, whereas k_2_ was the relaxation contribution coefficient. In Peppas-Sahlin model, when k_1_ > k_2_, the system follows a diffusion controlled mechanism, when k_1_ < k_2_, the system follows matrix controlled swelling mechanism. In this study PCL + CIL fibers followed a diffusion controlled mechanism. The modeling results showed that the Peppas-Sahlin equation fits the data well (R^2^= >0.983), and confirmed the diffusion/relaxation effect on CIL release from electrospun fibers. PCL is a hydrophobic polymer, and this characteristic could affect the penetration of aqueous medium through the electrospun material. After hydration, the interaction of fibrous polymer structure with the aqueous medium at 37°C could have led to polymer chain movement that allowed CIL to be released from polymer matrix via relaxation. The temperature set in this *in vitro* study was above the glass transition temperature of PCL (−60°C), which increased flexibility and mobility of polymer chain within electrospun fibers. Therefore, in given dissolution conditions, rearrangement of the polymer chains was possible and transport of a hydrophobic molecule through polymer phase might occurr.

Based on results obtained from *in vitro* release studies Ritger-Peppas and Peppas-Sahlin models provided the best fit for release profiles of CIL from electrospun PCL materials. According to Ritger-Peppas release exponent and Peppas-Sahlin contribution coefficients, drug dissolution in combination with diffusion and polymer relaxation were considered as the mechanisms for CIL release from the polymer matrix, since no significant evidence was reported for PCL degradation in such a short period of time (48 h) (74). As endothelial damage after graft or stent implantation is the critical factor behind neointimal hyperplasia or thrombosis (75), CIL release within subacute phase following device implantation and vascular injury would be beneficial to the reendothelialization process (76).

## Conclusions

Several PCL fibrous structures, with and without CIL, were prepared on a rotating drum collector and characterized for potential application as DDS in the cardiovascular system.

Electrospun structures exhibited near micron-sized fiber diameters from 0.77 to 2.97 μm depending on the polymer and drug concentration. The presence of a drug influenced average fiber diameter and fiber coherency. Despite the hydrophobic nature of drug and polymer, SEM analysis revealed the presence of surface crystallization, increasing with CIL concentration, which was probably related to the high solubility of CIL in chloroform. DSC thermographs indicated a decrease in enthalpy value which corresponded with an increase in CIL content, thus demonstrating successful CIL incorporation. The XRD results and the DSC data together show that collector rotation speed had no influence on the crystallinity of electrospun fibers. The FTIR and Raman spectra confirmed that CIL retained its crystalline A form structure after the electrospinning process.

The obtained nanomechanical properties of electrospun fibers matched those of collagen fibrils found in blood vessels. Drug release of CIL from electrospun PCL materials followed Ritger-Peppas and Peppas-Sahlin models and indicate diffusion-controlled release mechanism in combination with polymer relaxation. Additionally, release profile fell in the time-frame of subacute phase following device implantation and vascular injury, which could facilitate reendothelialization process. This demonstrates a very promising potential of electrospun drug delivering materials as medical devices for vascular tissue engineering and opens intriguing opportunities for scaffold-related applications in other fields of Medicine.

## Electronic supplementary material

ESM 1 (DOCX 7657 kb)

## ABBREVIATIONS

∆Hm: The enthalpy of melting derived from DSC curves
∆Hm_0_: The enthalpy of melting 100% crystalline polymer
λ: The X-ray wavelength
θ: The diffraction angle
API: Active pharmaceutical ingredient
ASA: Acetylsalicylic acid
BCS: Biopharmaceutical classification system
CIL: Cilostazol
D: Crystallite size
DDS: Drug delivery system
drug_obt_: The weight of the drug determined in obtained structure
drug_exp_: The weight of the drug expected in obtained structure
DSC: Differential scanning calorimetry
ECM: Extracellular matrix
ePTFE: Expanded polytetrafluroethylene
f_2_: Similarity factor
FTIR: Fourier transformed infrared spectroscopy
FWHM: Full width at half maximum
k: Shape factor
k_H_, k_HC_, k, k_1_, k_2_: Constants for Higuchi, Hixson-Crowell, Peppas-Sahlin and Ritger-Peppas models
M_∞_, M_0_: The starting amount of the active substance
m_1_: Weight of empty glass vessels
m_2_: Weight of vessels containing electrospun PCL tubes
m_3_: Weight of vessels containing electrospun PCL tubes after 24 h
m_exp_: Total sum of the masses of the polymer and drug used for the preparation of the structures
m_obt_: Mass of the obtained structure
M_t_: The cumulative amount of the active substance released at time t
n: The release exponent
PCL: Polycaprolactone
PET: Poly-ethylene terephthalate
PTX: Paclitaxel
SEM: Scanning electron microscopy
TEVG: Tissue-engineered vascular grafts
USP4: Flow-through cell apparatus
VSMC: Vascular smooth muscle cells
Xc: Crystallinity
