# Supplementary material for: Cilostazol-Loaded Poly(ε-Caprolactone) Electrospun Drug Delivery System for Cardiovascular Applications
Source: Pharm Res. 2018 Jan 16;35(2):32. doi: 10.1007/s11095-017-2314-0 (PMC5784006; doi:10.1007/s11095-017-2314-0)
Supplement: Supplementary file 1 — (DOCX 7657 kb) [file 11095_2017_2314_MOESM1_ESM.docx]

**Cilostazol-loaded poly(ε-caprolactone) electrospun drug delivery system for cardiovascular application**

Marek Rychter ^1,2*^, Anna Baranowska-Korczyc ^2^, Bartłomiej Milanowski ^1^, Marcin Jarek ^2^, Barbara M. Maciejewska ^2,3^, Emerson L. Coy ^2^, Janina Lulek ^1^

^1^Department of Pharmaceutical Technology, Faculty of Pharmacy, Poznan University of Medical Sciences, Grunwaldzka 6, 60-780 Poznań, Poland

^2^NanoBioMedical Center, Adam Mickiewicz University Poznan, Umultowska 85, 61-614 Poznań, Poland

^3^Department of Macromolecular Physics, Faculty of Physics, Adam Mickiewicz University, Umultowska 85, 61-614 Poznań, Poland

List of Figures:

- Average diameters and alignment coherencies of fibers from inner and outer layer of electrospun PCL tubular structures;
- Fiber orientation distribution presented as a color-coded map and histograms;
- DSC curves of tubular structures electrospun with various rotation speeds;
- XRD patterns of tubular structures electrospun with various rotation speeds.

**Table S1.** A comparison of the average diameters and alignment coherencies of fibers from inner and outer layer of electrospun PCL tubular structures collected on a rotating collector and a stationary collector.

**
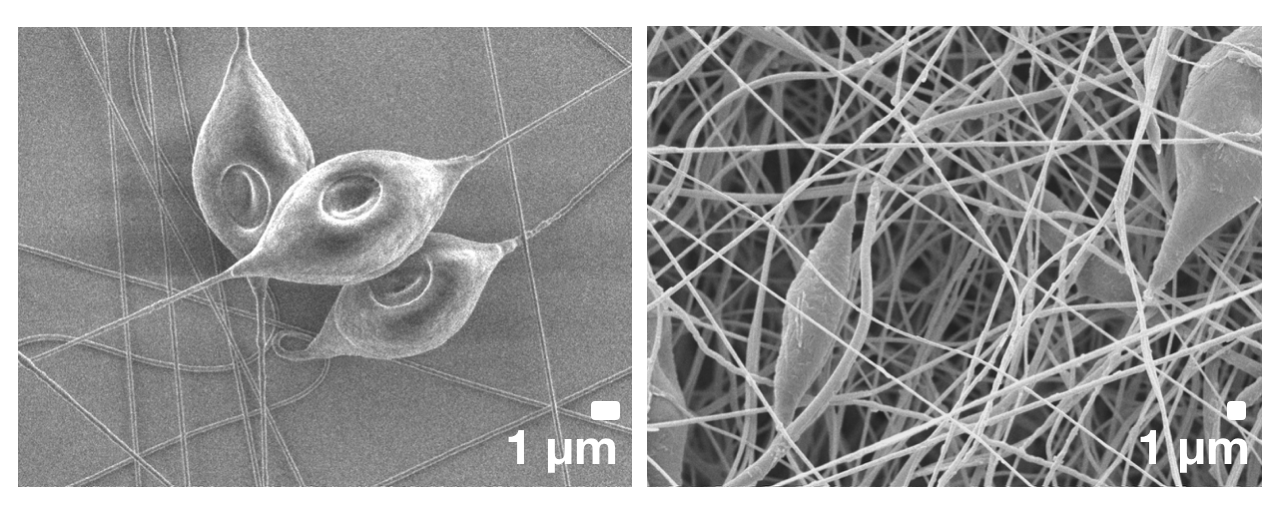
**

**Figure S1.** Electrospun 6% PCL formulation with beaded formations along fibers.

**

**

**Figure S2.** Fiber orientation presented as a color-coded SEM micrographs of various formulations of PCL fibers. Fiber orientation degree corresponds to a color from the bar presented at the bottom. Histograms demonstrating fiber orientation distribution are presented below each color-coded SEM micrograph. L and C refers to the longitudinal and circumferential directions of electrospun sample.

**
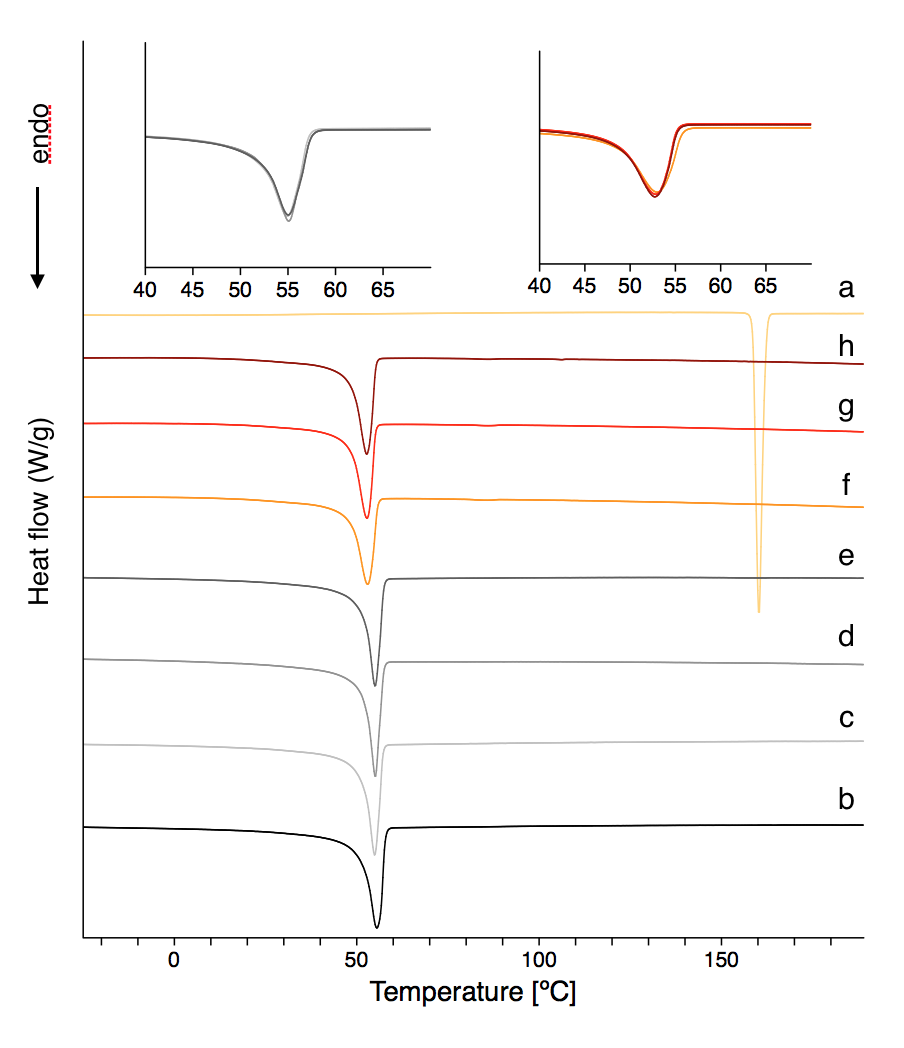
**

**Figure S3.** DSC curves of CIL powder (a), PCL pellets (b), PCL fibrous mats collected with various rotation speed: 1000 rpm (c), 2000 rpm (d), 4000 rpm (e), and PCL fibrous mats with 12.50% of CIL collected with various rotation speed: 1000 rpm (f), 2000 rpm (g), 4000 rpm (h). Insets represent stacked thermographs of of PCL fibrous mats (left) and PCL fibrous mats with 12.50% of CIL (right), visualizing no shifts in the melting temperature of PCL.

**
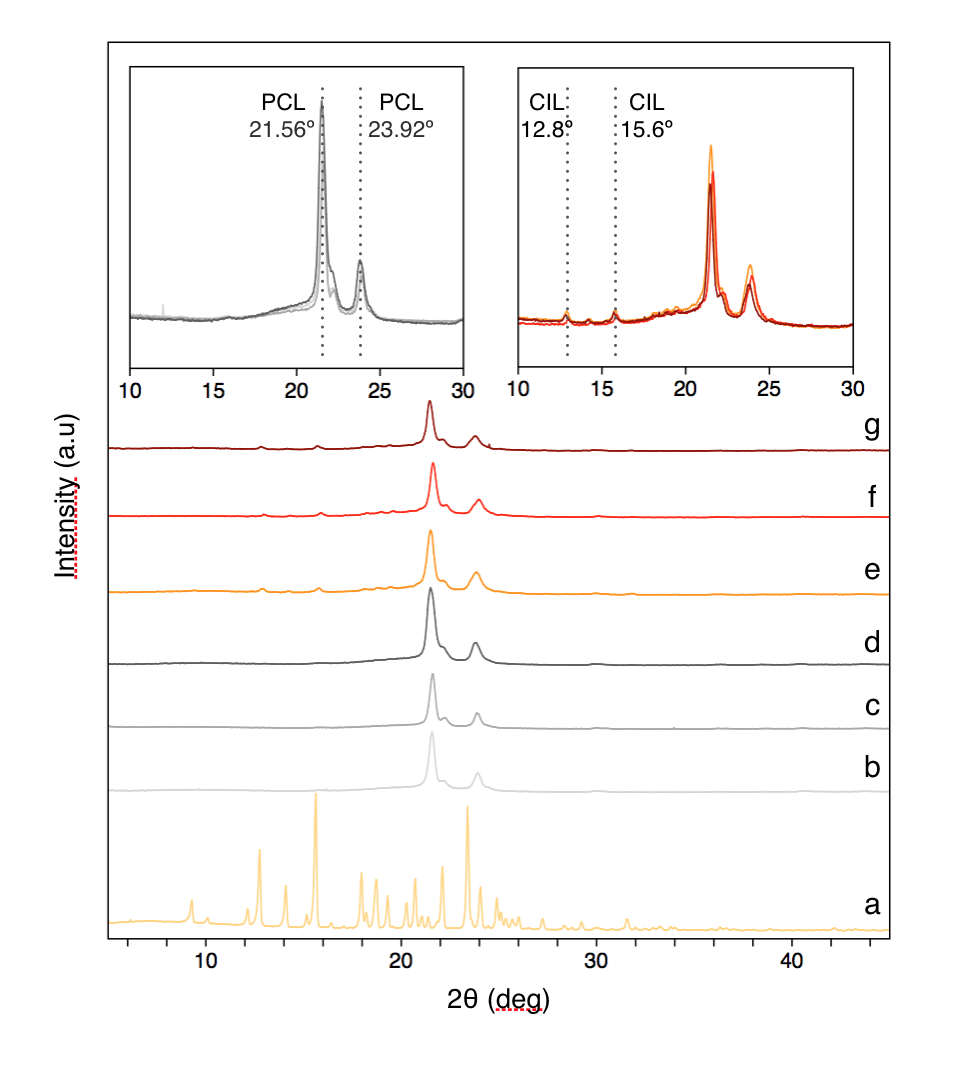
**

**Figure S4.** XRD patterns of CIL powder (a), PCL fibrous mats collected with various rotation speed: 1000 rpm (b), 2000 rpm (c), 4000 rpm (d), and PCL fibrous mats with 12.50% of CIL collected with various rotation speed: 1000 rpm (e), 2000 rpm (f), 4000 rpm (g). Insets represent stacked spectra of PCL fibrous mats (left) and PCL fibrous mats with 12.50% of CIL (right).
